# Visnagin treatment attenuates DSS-induced colitis by regulating inflammation, oxidative, stress, and mucosal damage

**DOI:** 10.3389/fvets.2025.1558092

**Published:** 2025-05-28

**Authors:** Vemula Sravathi, Madhuri Doppalapudi, Ravi Kumar Yadala, Anilkumar Banothu, Vijaya Kumar Anumolu, Hanuman Donga Durga Veera, Bhaskar Debbarma

**Affiliations:** ^1^Department of Veterinary Pathology, P.V. Narsimha Roa Telangana Veterinary University, Hyderabad, Telangana, India; ^2^Department of Pharmacology and Toxicology, P.V. Narsimha Roa Telangana Veterinary University, Hyderabad, Telangana, India; ^3^Department of Veterinary Public Health and Epidemiology, College of Veterinary Sciences, P.V. Narsimha Roa Telangana Veterinary University, Hyderabad, Telangana, India

**Keywords:** C57BL/6 mice, dexamethasone sodium, dextran sodium sulfate, disease activity index, oxidative stress, ulcerative colitis, visnagin

## Abstract

Ulcerative colitis (UC), is a chronic inflammatory bowel disease characterized by recurrent episodes of inflammation and ulceration of the colonic mucosa. This study aimed to explore the therapeutic potential effects of visnagin (VIS), a natural furanochromone using a murine model, focusing on tight junction protein expression, oxidative stress, apoptosis and associated inflammation in a dextran sodium sulfate (DSS) induced UC model. A total of 36 male C57BL/6 mice were divided randomly into six groups (*n* = 6): Group 1 served as the control, group 2, treated with DSS (2% with three 5-day cycles diluted in distilled water administered orally). Group 3 (VIS) *perse* alone (60 mg/kg b. wt), orally for 31 days, Group 4-low dose of VIS (30 mg/kg b. wt for 31 days with DSS, group 5-high dose VIS (60 mg/kg b. wt) for 31 days with DSS and Group 6 Dexamethasone sodium @ 1 mg/kg b. wt-IP with DSS for 31 days. Disease progression and therapeutic outcomes were assessed by monitoring clinical symptoms, body weight changes, colon length, Disease activity index (DAI), oxidative stress indices, gross and histopathological analysis, inflammatory cytokine levels and immunohistochemical expression. Results demonstrated that VIS co-administration, particularly at high doses, significantly mitigated DSS-induced weight loss, colon shortening. This protective effect was further supported by a significant reduction in oxidative and nitrosative stress which was evident from decreased levels of nitrite and Malondialdehyde (MDA) in VIS treated groups 4 and 5. Further, VIS suppressed pro-inflammatory cytokines (TNF-*α*, IL-1*β*, IL-6, IFN-*γ*, NF-κB, IL-17, MPO and TGF-β) while increasing anti-inflammatory IL-10 levels in colon tissues. Reverse transcription polymerase chain reaction (RT-PCR) analysis revealed significantly reduced mRNA expression of TNF-*α* and IL-17 along with increased occludin expression in groups 4, 5 and 6. VIS also improves intestinal barrier by increasing the expression of tight junction occludin, as confirmed through RT-PCR. Immunohistochemical analysis showed strong positive immunoreactivity for NF-κB, COX-2, NLRP3 and TNF-*α* in DSS group, which wa notably reduced in VIS-treated groups. Additionally, VIS improved intestinalbarrier integrity by upregulating occluding expression. Histopathological analysis further confirmed that VIS attenuated DSS-induecdcolonic lesions. In conclusion, VIS exhibits potent anti-inflammatory and mucosal-protective properties, making it a promising therapeutic candidate for managing UC. Its ability to modulate inflammatory pathways and enhance intestinal barrier function suggests its potential as an alternative treatment for UC.

## 1 Introduction

Ulcerative colitis (UC), a type of inflammatory bowel disease (IBD), is characterized by chronic inflammation of the gastrointestinal tract (1). Currently, millions of people worldwide suffer from UC, which significantly increases the risk of developing colorectal cancer, one of the third most prevalent cancers globally (2). Prevalence of UC is steadily increasing in developing countries largely due to the adoption of Western lifestyles, including dietary shifts (3). While the exact cause of UC remains unclear, it is widely believed that a combination of genetic predisposition, environmental factors such as diet, alcohol consumption, smoking and obesity contribute to its development (4). In addition, Oxidative stress and inflammatory responses play key roles in the pathogenesis of UC (5). As a result, enhancing antioxidant and anti-inflammatory capabilities has become a central focus in current research aimed at preventing the progression of the disease (6). Over the last few decades, numerous animal models have been developed to gain knowledge on the complexity of IBD pathogenesis, delineating underlying molecular mechanisms.

Rodent models of UC play a crucial role in the advancement of new therapeutic approaches for IBD. The most frequently used mouse model of colitis employs dextran sodium sulfate (DSS), a chemical colitogen with anticoagulant properties, because of easy to administer through drinking water effectively mimics human UC, and provides valuable insights into its pathogenesis (7). DSS breaks epithelial barrier, exposing lamina propria to antigens and bacteria. This model is simple to establish, fast, reliable, strong and highly reproducible (8). While the exact causes of UC are not fully understood, growing evidence suggests that the disease is strongly linked to inflammation, oxidative stress imbalance, intestinal barrier disruptions and gut microbiota abnormalities. Nuclear factor-kappa B (NF-κB) is a key transcriptional regulator in the inflammatory process, typically remaining inactive due to its binding with inhibitor kappa B (IκB) (9). When activated by certain stimuli, NF-κB triggers the expression of various genes and cytokines, contributing to the inflammatory response and maintaining physiological functions. However, excessive activation of NF-κB can lead to autoimmune conditions, including UC (10). Similarly, Nuclear factor erythroid 2-related factor 2 (Nrf-2) is a crucial transcription factor involved in combating oxidative stress. Under normal conditions, Nrf-2 is bound to Kelch-like ECH-associated protein 1 (Keap1) *via* E3 ubiquitin ligase, leading to its degradation through ubiquitination. During oxidative stress, Nrf-2 is activated, causing it to detach from Keap1, move into the nucleus and promote the transcription of antioxidant enzymes. This mechanism makes Nrf-2 central to antioxidative defense. Existing drug therapies primarily aim to extend the period of clinical remission and delay the progression and severity of the disease. Studies have shown reduced Nrf-2 expression in DSS-induced UC models (11), suggesting that drugs capable of activating Nrf-2 could be promising for UC treatment.

Visnagin {(VIS) [4-methoxy-7-methyl-5-furo (3, 2)-benzopyran-5-one]} is an active furanocoumarin derivative found in *Ammi visnaga* plant known for its antioxidant and anti-inflammatory actions. Its unique structure, which incorporates a methoxy group at the 5-position and a methyl group attached to the furan ring, contributes to its pharmacological activity (12). VIS exhibits favorable pharmacokinetics, good bioavailability, and a safety profile conducive to therapeutic use (13). VIS has shown protective effects against cerulein-induced acute pancreatitis in mice by enhancing the expression of Nrf2, a key regulator of antioxidant defense mechanisms, while concurrently suppressing the activity of NF-κB signaling pathway (14). Furthermore, VIS exhibits anti-inflammatory properties against kainic acid-induced neuronal injury and apoptosis (15). Recently, studies have shown that VIS attenuated inflammation and oxidative tissue injury in the testes and cardiac injury by upregulating Nrf2 signaling in rats (16). Additionally, VIS inhibited the release of inflammatory cytokines in pulmonary tissue (17). However, the impact of VIS on DSS-induced chronic colitis has not yet been studied. Therefore, our research aimed to assess the potential therapeutic effects of VIS in mice with recurrent DSS-induced chronic ulcerative colitis and to explore the mechanisms behind its action.

## 2 Materials and methods

### 2.1 Chemical source

Dextran sulfate sodium salt 500 was procured from Savvy Scientifics (CAT No. 99629)., Visnagin was procured from Cayman Chemical Company, United States (CAT No. 34140), Stains and chemicals for the histopathological study of tissues were obtained from HiMEDIA Laboratories Pvt. Ltd., Secunderabad, Telangana. Antibodies of NF-κB, occludin, TNF-*α*, Nrf2, NLRP3, COX-2 and GFAP for immunohistochemical staining were procured from M/s Santacruz Biotechnology, United States. PolyExcel HRP/ DAB detection system for immunohistochemistry was procured from M/s PathnSitu Biotechnologies, United States.

### 2.2 Experimental design

A total of 36 male C57BL/6 mice (weighing 25-30gms, aged 6–8 weeks) were obtained from the M/S Jeeva Life Sciences Ltd. Hyderabad and maintained in a controlled environment throughout the experiment.

#### 2.2.1 Ethical approval

The experimental protocol was reviewed and approved by Institutional Animal Ethics Committee, C.V.Sc., Rajendranagar, Hyderabad (06/26/CVSc, Hyd. IAEC/2023).

Animals were maintained under hygienic environmental conditions in polypropylene cages at 22–24°C, 12 h (h) of a light- dark cycle (12:12 and 40–70% of relative humidity). Mice were provided with 7 days of acclimation period before the experiment began and allowed free access to sterile pellet food and *ad libitum* drinking water. To assess the ameliorative effects of visnagin on colon caused by DSS induced ulcerative colitis, these mice were randomly assigned to six experimental groups (*n* = 6).

**Figure 1:**
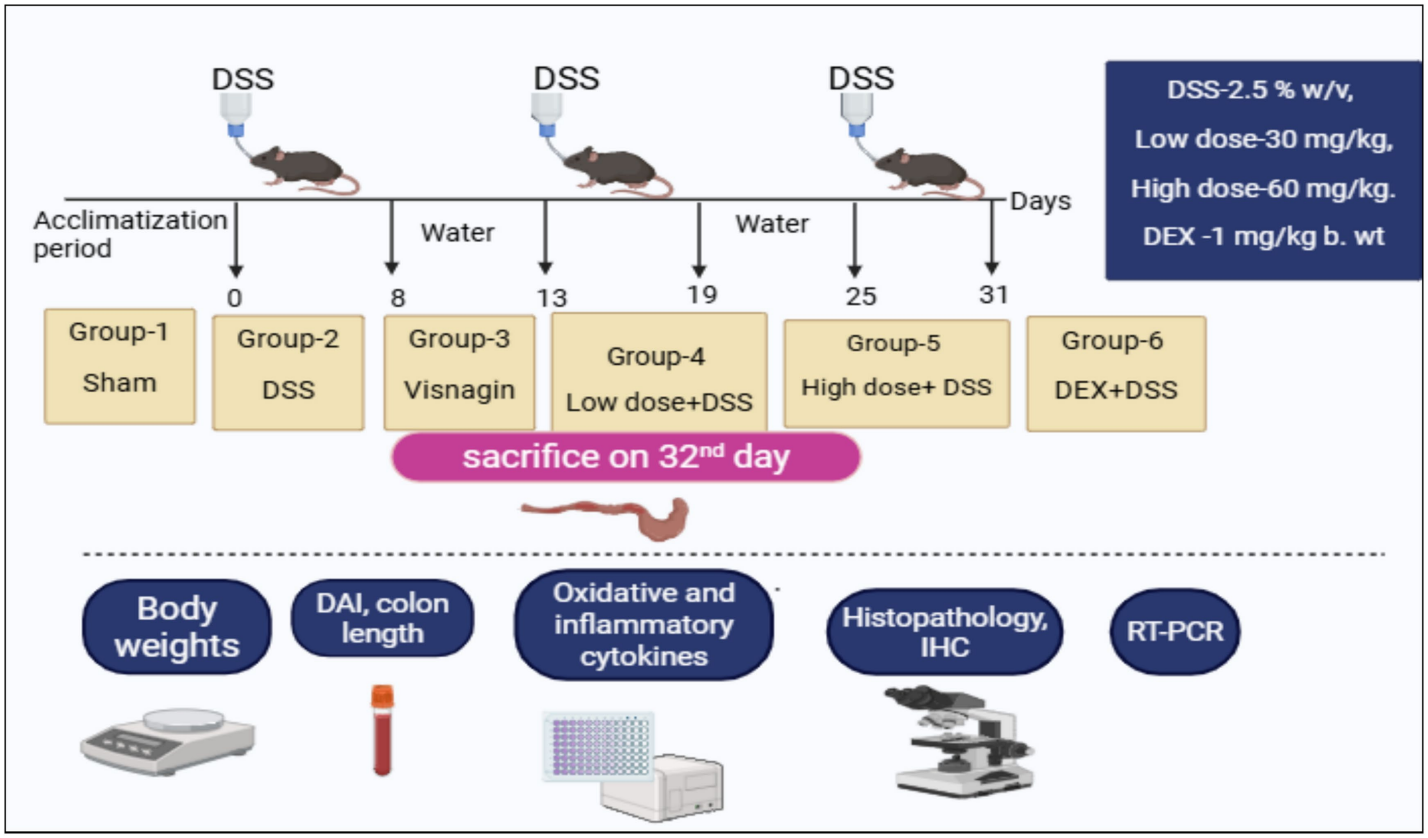
Experimental design of the current study.

Experimental mice were closely monitored to detect any changes in behavior signs. Key physiological indicators, including body weight, body temperature and dehydration levels, were regularly checked to safeguard their well-being. Any unusual activity patterns or signs of discomfort were immediately noted, addressed and documented. The experimental design adopted for the present study (Table 1 and Figure 1) (12, 18).

**Table 1:** Experimental design.

Group	No. of mice	Treatments
Group 1	6	Sham-Normal saline for 31 days
Group 2	6	DSS (@ 2% with 3 cycles interval with 5 days diluted in distilled water Per orally)
Group 3	6	Visnagin *perse* @ 60 mg/kg b.wt for 31 days Per orally
Group 4	6	Low dose: Visnagin @ 30 mg/kg b. wt for 31 days + DSS (@ 2% with 3 cycles interval with 5 days), Per orally
Group 5	6	High dose: Visnagin @ 60 mg/kg b. wt for 31 days + DSS (@ 2% with 3 cycles interval with 5 days), Per orally
Group 6	6	DSS (@ 2% with 3 cycles interval with 5 days) + Dexamethasone sodium (@ 1 mg/kg b. wt- IP) for 31 days

### 2.3 Sampling

At the end of the experiment (32nd day), following blood collection, mice were euthanized with carbon dioxide (CO_2_) using a CO_2_ chamber, and a detailed necropsy examination was carried out as per the standard procedure suggested by Cuzic et al. (19). The colon tissue was dissected, the colon length was measured, and gross lesions in the colon were recorded. In suitable preservative, small slices of respective tissue samples were collected for histopathology, immunohistochemistry (IHC) and ultrastructural pathology (colon). Small pieces of the colon were also collected and stored at −20°C to study oxidative, antioxidant, and inflammatory cytokine profiles.

### 2.4 Body weights

Body weights of all the mice were recorded on day 1 and subsequently at weekly intervals of the experiment.

### 2.5 Assessment of disease activity index

A DAI was used to evaluate the intensity of intestinal damage. An individual scoring system was employed to determine the DSS-induced insult, as per Zhao et al. (20). The DAI was assessed based on the parameters mentioned below (Table 2).

**Table 2:** Assessment of disease activity index score (20).

Parameter	Observation	Score
Percentage loss in body weight (%)	No loss 1–5% 6–10% 1–20% ≥21%	0 1 2 3 4
Stool consistency	Normal stool Loose stool Watery diarrhea	0 1–2 3–4
Presence of bloody stool	No bloody stool Slight bleeding Excessive bleeding	0 1–2 3–4

### 2.6 Estimation of oxidative stress indices

Colon tissue was collected and stored in liquid nitrogen (−80°C) for subsequent analysis of oxidative stress markers like thiobarbituric acid reactive substances (TBARS), nitrite levels, as well as antioxidant indices like glutathione (GSH), Catalase (CAT), and Superoxide dismutase (SOD) enzymes. TBARS and nitrite levels help to indicate oxidative and nitrosative stress of colon tissues. One gram of colon tissue was homogenized in Tris HCl buffer (10 mL, pH 7.2). The homogenate was then centrifuged for 2 min for biochemical assays. The concentration of lipid peroxidation activity (LPO) was evaluated using standard TBARS protocol (21), while nitrosative stress was measured using Griess reagent (22). Reduced GSH assay was estimated using Ellman’s procedure (23), SOD levels by mixing pyrogallol and MTT reagent (24, 25), and Catalase levels were estimated by adding H_2_O_2_ and 2 μL of dichromate acetic acid and heat for 10 min to know antioxidant profile (25).

### 2.7 Estimation of cytokine profile

An enzyme-linked immune sorbent assay (ELISA) kit was used to know specific cytokine inflammatory biomarkers procured from Krishgen biosystems, Mumbai. Approximately 100 mg of colon tissue was homogenized with Tris-Triton lysis buffer and centrifuged at 10,000 rpm for 10 min. A 96-well ELISA plate was prepared by coating each well with 100 μL of primary antibody solution, followed by overnight incubation. Then the wells were washed with wash buffer five times, adding 200 μL of blocking buffer for 1 h, and followed plates were washed four times. Subsequently, 100 μL of samples and standards were added and incubated for 2 h at room temperature. After the incubation, the samples were discarded, and 100 μL of detection antibody was added to each well, followed by a 1-h incubation at room temperature. The plates were washed four times with 200 μL of wash buffer and added 100 μL/well of Streptavidin-HRP solution. The plates were incubated for 20 min in the dark, then washed seven times with 200 μL of wash buffer. Finally, 100 μL of TMB substrate solution was added to each well and incubated for 15 min at room temperature. The reaction was stopped by adding 1 M H3PO4 stop solution to each well, and the absorbance was measured at 450 nm after color developed, and results were expressed in units pg./mg of protein.

### 2.8 Myeloperoxidase activity assay

MPO activity is an indicator of neutrophil infiltration in ulcerative colitis. Colon tissues were homogenized in ice-cold 0.1 M potassium phosphate buffer (pH 6.5) containing 0.5% hexadecyl trimethyl ammonium bromide (HTAB) and 10 mM EDTA. The obtained tissue homogenates were centrifuged for 20 min at 4°C. An aliquot of the supernatant (0.1 mL) was mixed with 2.9 mL of 50 mM phosphate buffer containing 0.05% hydrogen peroxide. The change in absorbance over 5 min was measured at 460 nm. The results were expressed as U/g of tissue (26).

### 2.9 Gross and histopathological studies

Six mice from each group were sacrificed on the 32nd day of the experimental period to study gross and histopathology. Detailed necropsy was conducted, and gross lesions, if any, were recorded. Representative colon samples were collected in 10% neutral buffered formalin (NBF) and fixed. Samples were processed, sectioned (5 μm), and stained with Hematoxylin and Eosin (H&E) as per the standard protocol (27).

### 2.10 Immunohistochemistry

Immunohistochemical analysis was carried out on formalin-fixed, paraffin-embedded sections of colon tissue with a thickness of 4 μm. In brief, the tissue sections were incubated overnight at 4°C with primary antibodies targeting NF-κB, occludin, TNF-*α*, Nrf2, NLRP3, and COX-2. Afterward, the sections were rinsed with phosphate-buffered saline (PBS) and treated with a secondary antibody. Then, it was treated with Diaminobenzidine (DAB), counterstained with hematoxylin, and observed under optical microscope at 100 × magnification.

### 2.11 Ultrastructural pathology

Soon after sacrifice, thin slices of the colon were dissected and fixed in 2.5% glutaraldehyde (Sigma Aldrich, United States) in 0.1 M phosphate buffer (pH 7.3), washed in buffer, and fixed in 1% aqueous osmium tetroxide (Sigma Aldrich, USA) for 2 h then dehydrated in ascending grades of acetone (Qualigens fine chemicals, Mumbai) and subjected to vacuum desiccation for 45 min and mounted over stubs on the double-sided carbon conductivity tape and uniform sputtering of gold was done with sputter coater (JEOL-JFC-1600) for 180 s as per the standard protocol of Ruska Labs (28). Later specimens were observed under a Scanning Electron Microscope (JEOL; JSM-5600, Japan).

### 2.12 Real time-polymerase chain reaction

Concurrently, slices of colon tissues were procured for the assessment of inflammation and oxidative markers (Occludin, TNF-*α* and IL-17) through RT-PCR technique. Total RNA isolation from colon tissues was executed with TRIzol reagent, following guidelines of manufacturer. Subsequently, cDNA synthesis was carried out, utilizing 1 μg of total RNA, employing a Reverse Transcriptase M-MLV Kit. The amplification cycling conditions encompassed an initial denaturation step at 95°C for 10 min, succeeded by 40 cycles of denaturation at 95°C for 15 s and annealing/extension at 60°C for 1 min. Housekeeping gene GAPDH served as the internal control and the relative threshold cycle values (ΔCt) were utilized for analyzing mRNA expression levels (Table 3) (29).

**Table 3:** Primer sequences used for the standardization protocol of RT-PCR.

S. No.	Gene	Forward primer sequence	Reverse primer sequence
1.	GAPDH	5′TGGAGAAACCTGCCAAGTATGA-3′	5′TGGAAGAATGGGAGTTGCTGT-3′
2.	Occludin	5′ACGGACCCTGACCACTATGA-3′	5′TCAGCAGCAGCCATGTACTC-3′
3.	IL-17	5′TTTAACTCCCTTGGCGCAAAA-3′	5′CTTTCCCTCCGCATTGACAC-3′
4.	TNF-α	5′CTCATGCACCACCATCAAGG-3′	5′ACCTGACCACTCTCCCTTTG-3′

### 2.13 Statistical analysis and its interpretation

The findings from current experiments are reported as mean±SE values. Statistical analysis was performed using GraphPad Prism version 9.0 software (GraphPad Software, CA, United States), which was exposed to a one-way analysis of variance (ANOVA) applying Tukey’s multiple comparison test. A *p-*value of less than 0.001 was considered statistically significant in every group (54).

## 3 Results

### 3.1 Visnagin relieves clinical signs of UC

**Figure 2:**
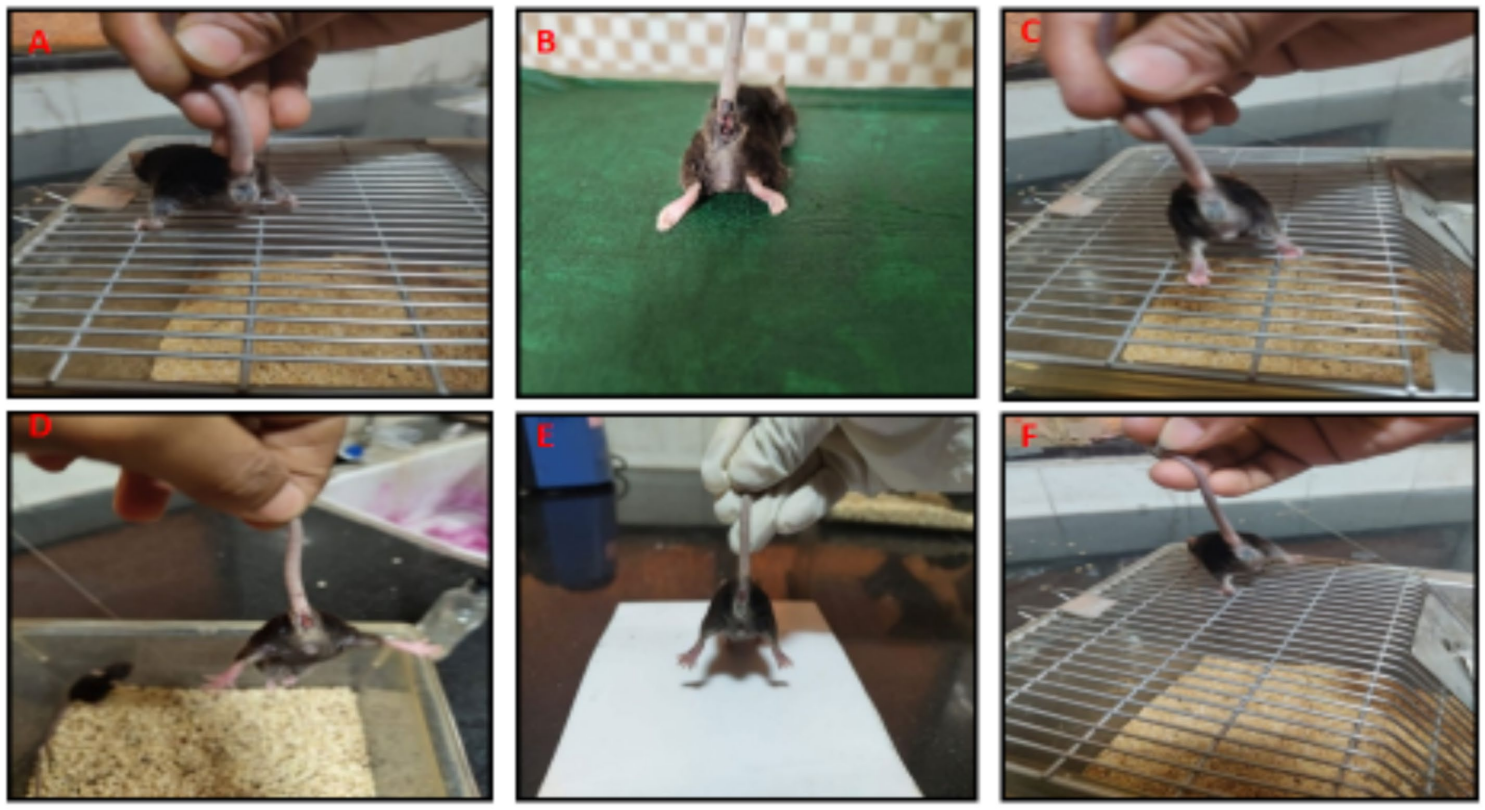
Gross appearance of rectum from 6 groups. Mice showing normal appearance of rectum (G-1,3, **A,C**), severe rectal hemorrhages (G-2, **B**), moderate rectal hemorrhages (G-4, **D**), mild rectal hemorrhages (G-6, **E**), mild rectal hemorrhages (G-6, **F**).

The experimental mice in group 1 and group 3 showed no characteristic symptoms and remained apparently healthy throughout the study (Figures 2A–C). The mice in group 2 showed signs of reduced feed and water intake, decreased body weight gain and bloody, watery diarrhea from the 4th day onwards (Figure 2B). Compared to the model group, mice in groups 4, 5, and 6 showed mild watery diarrhea with mild clinical signs suggesting an ameliorative effect of VIS on UC (Figures 2D–F).

### 3.2 Ameliorative effect of VIS on body weight gain

**Figure 3:**
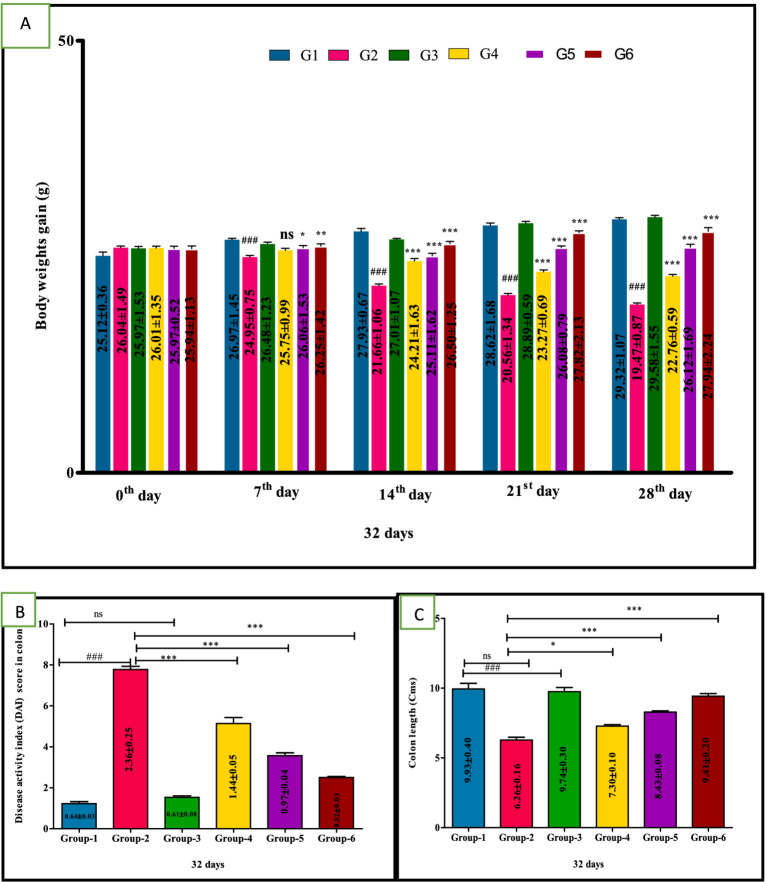
Weekly body weights (g) **(A)**, Disease activity index (DAI) score in colon **(B)**, Colon length **(C)** in different groups of mice. Statistical analysis was done with one-way ANOVA using Tukey’s multiple comparison test (graph pad vision version). Means with *, # as superscripts differ significantly among groups. ^###^*p* < 0.001, *p < 0.1, **p < 0.01 G-2 Vs G-4, ***p < 0.001.

There was a significant (*p* < 0.05) reduction in body weight in the DSS model group 2 compared to other treatment groups. In contrast, the body weight mean values of treatment group 4 mice showed a non-significant increase on the 7th day and a significant (*p* < 0.001) increase from the 2nd to 4th week. Mice in groups 5 and 6 exhibited significant improvements (*p* < 0.001) from 1st to 4th week compared to group 2. These findings suggest VIS administration significantly mitigates the reduction in body weights associated with DSS-induced UC (Figure 3A). The mean values in group 3 were comparable to group 1, and values are depicted below (Table 4).

**Table 4:** Weekly body weights (g) in different groups of mice.

Groups	Day 0	Day 7	Day 14	Day 21	Day 28
Group 1	25.12 ± 0.36	26.97 ± 1.45	27.93 ± 0.67	28.62 ± 1.68	29.32 ± 1.07
Group 2	26.04 ± 1.49	24.95 ± 0.75^###^	21.66 ± 1.06^###^	20.56 ± 1.34^###^	19.47 ± 0.87^###^
Group 3	25.97 ± 1.53	26.48 ± 1.23	27.01 ± 1.07	28.89 ± 0.59	29.58 ± 1.55
Group 4	26.01 ± 1.35	25.75 ± 0.99^ns^	24.21 ± 1.63***	23.27 ± 0.69***	22.76 ± 0.59***
Group 5	25.97 ± 0.52	26.06 ± 1.53*	25.11 ± 1.62***	26.08 ± 0.79***	26.12 ± 1.69***
Group 6	25.94 ± 1.13	26.25 ± 1.42*	26.50 ± 1.26***	27.82 ± 2.13***	27.94 ± 2.24***

^###^*p* < 0.001 G-1 Vs G-2, *p < 0.05 G-2 Vs G-4, ***p < 0.001 G-2 Vs G-4.

### 3.3 Ameliorative effect of VIS on disease activity index score in colon

The DSS treatment group 2 showed significantly (*p* < 0.001) higher mean values of DAI, compared to other groups. Whereas significant (*p* < 0.01, *p* < 0.001 and *p* < 0.001, respectively) improvement in DAI of treated groups 4, 5 and group 6 compared to DSS group 2. Additionally, group 5 mice showed a significant (*p* < 0.05) improvement compared to group 4 in a dose-dependent manner (Figure 3B). The mean values in group 3 were comparable to group 1, and values are depicted below (Table 4).

### 3.4 Effect of VIS colon length (Cms)

A significant (*p* < 0.001) decrease in the colon length of group 2 was observed compared to control group 1 mice. In contrast, colon length was significantly (*p* < 0.01, *p* < 0.001, and *p* < 0.001, respectively) increased in the VIS-treated groups 4, 5, and standard-treated group 6 when compared to DSS-treated group 2. Additionally, group 5 mice showed a significant (*p* < 0.05) improvement compared to group 4 in a dose-dependent manner. Furthermore, a significant (*p* < 0.05) difference was also observed between group 5 and group 6 (Figure 3C). The mean values in group 3 were comparable to group 1, and values are depicted (Table 5).

**Table 5:** Effect of visnagin in DAI score, colon length, oxidative, and antioxidant indices in colon of different groups of mice.

Groups	DAI	Colon length	TBARS-nM/mg protein	Nitrite (μm/mg protein)	SOD-IU/mg protein	GSH-μM/mg protein	Catalase (IU/mg protein)	MPO (U/gm protein)
Group 1	1.22 ± 0.10	9.93 ± 0.40	11.10 ± 1.65	4.99 ± 0.15	24.75 ± 1.71	42.56 ± 3.30	10.17 ± 0.73	0.43 ± 0.03
Group 2	7.11 ± 0.50^###^	6.26 ± 0.16^###^	46.73 ± 2.80^###^	34.26 ± 0.56^###^	5.47 ± 0.60^###^	15.12 ± 1.06^###^	2.85 ± 0.36^###^	1.38 ± 0.03^###^
Group 3	1.35 ± 0.06	9.74 ± 0.30	12.96 ± 2.12	5.25 ± 0.07	24.09 ± 1.24	40.33 ± 3.70	8.97 ± 0.85	0.41 ± 0.03
Group 4	5.13 ± 0.30**	7.30 ± 0.10*	31.74 ± 3.00*	20.90 ± 0.38***	16.26 ± 0.78***	26.28 ± 0.80*	6.04 ± 0.43*	1.02 ± 0.05***
Group 5	3.65 ± 0.14***	8.43 ± 0.08***	21.73 ± 2.20***	13.91 ± 0.83***	20.81 ± 0.90***	32.46 ± 1.80***	7.99 ± 0.65***	0.81 ± 0.04***
Group 6	2.50 ± 0.05***	9.41 ± 0.20***	18.76 ± 2.07***	9.03 ± 0.26***	21.17 ± 1.93***	33.82 ± 1.30***	8.92 ± 0.59***	0.64 ± 0.02***

^###^*p* < 0.001 G-1 Vs G-2, *p < 0.05 G-2 Vs G-4, **p < 0.01 G-2 Vs G-4, ***p < 0.001 G-2 Vs G-4.

### 3.5 Anti-oxidative effect of VIS on oxidative stress indices

**Figure 4:**
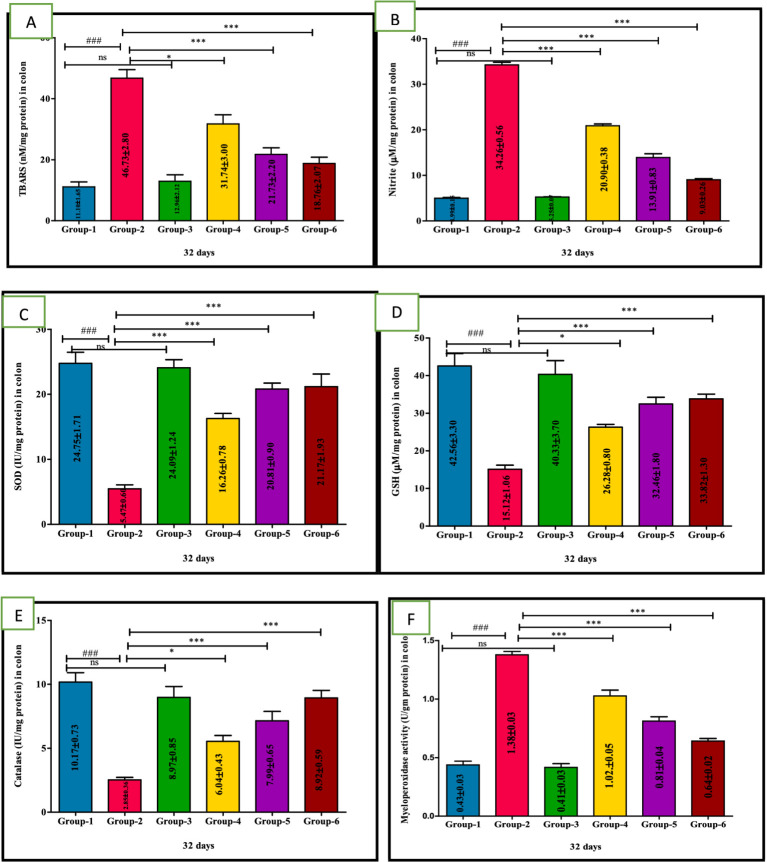
Ameliorative effect of Visnagin on oxidative stress parameters TBARS **(A)**, Nitrite **(B)**, SOD **(C)**, GSH **(D)**, Catalase **(E)**, Myeloperoxidase activity in different groups of colon **(F)**. Statistical analysis was done with one-way ANOVA using Tukey’s multiple comparison test (graph pad vision version). Means with *, # as superscripts differ significantly among groups. ^###^*p* < 0.001, *p < 0.05 G-2 Vs G-4, **p < 0. 01, ***p < 0.001.

Group 2 exhibited a significant (*p* < 0.001) elevation in the TBARS and nitrite levels compared to group 1. Interestingly, significant (*p* < 0.001) reduction in the TBARS and nitrite values in groups 4, 5, and group 6 compared to group 2 mice were observed. Furthermore, TBARS and nitrite concentration in group 5 did not significantly differ from the mean values observed in standard group 6. The control group 1 and the sham group 3 also showed no significant variation, and their mean values were similar (Table 5 and Figures 4A,B).

The levels of GSH, SOD and CAT significantly (*p <* 0.001) lowered in the model DSS group 2. However, compared with group 2, GSH, SOD and CAT levels were significantly increased in the treatment groups 4, 5, and 6 indicating a potential ameliorative effect against oxidative stress. Additionally, group 5 showed significantly (*p* < 0.05) greater improvement than group 4 in a dose-dependent manner. Notably, no significant difference in GSH activity was observed between group 5 and group 6 nor between *per se* group 3 and the untreated group 1 (Figures 4C–E); values are depicted (Table 5).

### 3.6 Ameliorative effect of VIS on myeloperoxidase activity

The MPO activity in DSS-treated group 2 was significantly (*p* < 0.001) higher compared to other groups. Conversely, mice treated with VIS in groups 4, 5, and the standard group 6 showed significant (*p* < 0.001) improvement compared to group 2 mice (Figure 4F), and values are depicted (Table 5).

### 3.7 Ameliorative action of VIS on inflammatory cytokines storm in UC

**Figure 5:**
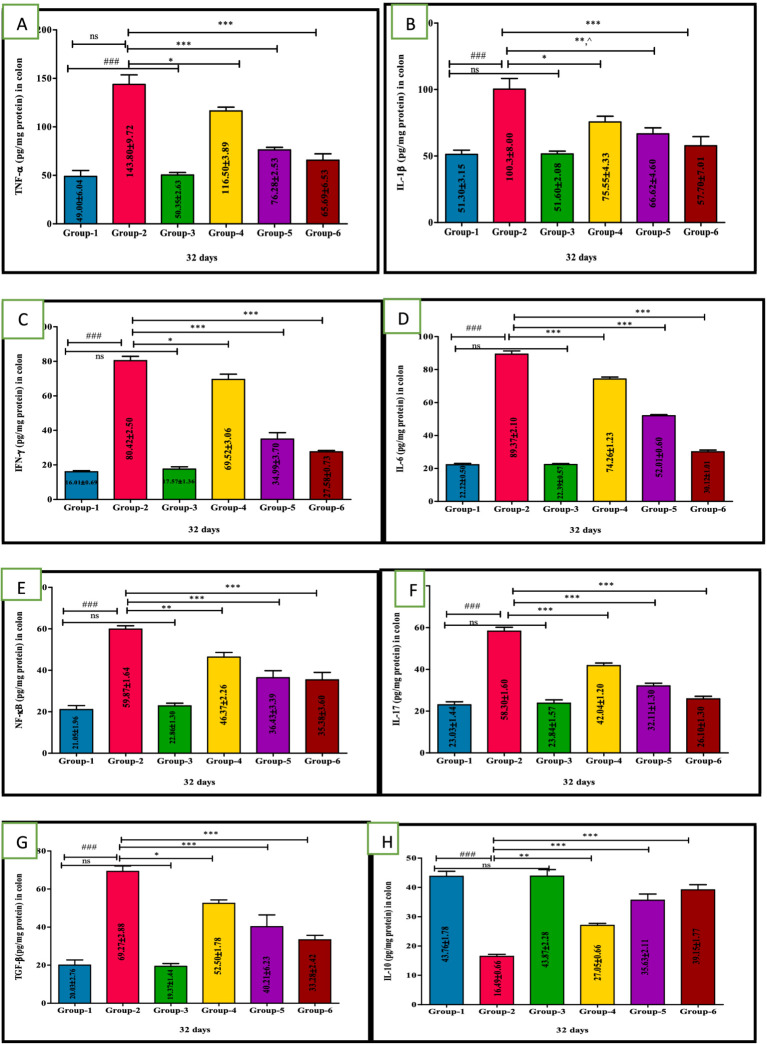
Ameliorative effect of Visnagin on inflammatory cytokine TNF-*α* **(A)**, IL-1*β* **(B)**, IFN-*δ* **(C)**, IL-6 **(D)**, NF-κB **(E)**, IL-17 **(F)**, TGF-β **(G)**, IL-10 **(H)**. groups. ^###^*p* < 0.001 G-1 Vs G-2, *p < 0.05 G-2 Vs G-4, **p < 0.01 G-2 Vs G-4, ***p < 0.001 G-2 Vs G-4.

A significant (*p* < 0.001) higher mean values of TNF-*α*, IL-1β, IL-6, IFN-*γ*, and NF-κB and a statistically significant (*p* < 0.001) reduced levels were recorded in group 2 compared to other groups due to immune-mediated inflammatory pathway. In contrast, VIS-treated groups 4, 5, and standard group 6 showed significantly (*p* < 0.05, *p* < 0.01, and *p* < 0.001, respectively) reduced mean levels and increased IL-10 levels compared to group 2 in a dose-dependent manner, indicating anti-inflammatory properties of VIS through decreasing proinflammatory effects and enhancing IL-10 anti-inflammatory levels. However, group 5 exhibited non-significant mean values compared to group 6. No significant disparity was noticed between groups 1 and 3 mice (Figures 5A–H) and values are depicted (Table 6).

**Table 6:** Effect of visnagin in pro-inflammatory and anti-inflammatory cytokine indices in colon of different groups of mice.

Groups	TNF-α-pg/mg protein	IL-1β-pg/mg protein	IFN-γ-pg/mg protein	IL-6-pg/mg protein	NF-κB-pg/mg protein	IL- 17-pg/mg protein	TGF-β-pg/mg protein	IL-10-pg/mg
Group 1	49.00 ± 6.04	51.30 ± 3.15	16.01 ± 0.69	22.22 ± 0.50	21.05 ± 1.96	23.45 ± 1.52	20.03 ± 2.76	43.76 ± 1.78
Group 2	143.80 ± 9.72^###^	100.30 ± 8.00^###^	80.42 ± 2.50^###^	89.37 ± 2.10^###^	59.87 ± 1.64^###^	57.72 ± 2.11^###^	69.27 ± 2.88^###^	16.49 ± 0.66^###^
Group 3	50.35 ± 2.63	51.60 ± 2.08	17.57 ± 1.36	22.39 ± 0.57	22.86 ± 1.30	24.82 ± 1.40	19.37 ± 1.44	43.87 ± 2.28
Group 4	116.50 ± 3.89*	75.55 ± 4.33*	69.52 ± 3.06*	74.26 ± 1.23***	46.37 ± 2.26**	44.86 ± 1.35*	52.50 ± 1.78*	27.05 ± 0.66**
Group 5	76.28 ± 2.53***	66.62 ± 4.60**,^	34.99 ± 3.70***	52.01 ± 0.60***	36.43 ± 3.39***	38.54 ± 1.58***	40.21 ± 6.23***	35.63 ± 2.11***
Group 6	65.69 ± 6.53***	57.70 ± 7.01***	27.58 ± 0.73***	30.12 ± 1.01***	35.38 ± 3.60***	34.05 ± 1.80***	33.28 ± 2.42***	39.15 ± 1.77***

^###^*p* < 0.001 G-1 Vs G-2, *p < 0.05 G-2 Vs G-4, **p < 0.01 G-2 Vs G-4, ***p < 0.001 G-2 Vs G-4.

### 3.8 Ameliorative action of VIS on expression of tight junction protein and inflammatory proteins in UC

The RT-PCR was performed to explore the expression of occludin, IL-17 and TNF-*α* against DSS-induced UC and to assess the protective effects of VIS. RT-PCR analysis of colon tissue revealed significant changes in the gene expression of occludin, IL-17, and TNF-*α* across experimental groups, highlighting the effects of DSS and the protective role of VIS.

**Figure 6:**
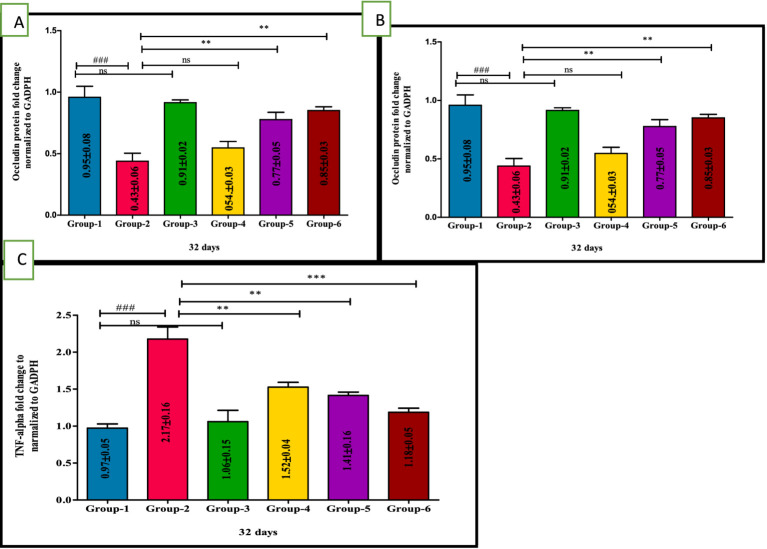
Ameliorative effect of Visnagin on gene expression of inflammatory cytokines Occludin **(A)**, IL-17 **(B)**, TNF-α **(C)**. ^###^*p* < 0.001 G-1 Vs G-2, **p < 0.01 G-2 Vs G-4, ***p < 0.001 G-2 Vs G-4.

Occludin expression, critical for intestinal barrier integrity, was significantly reduced in group 2 compared to all other groups. Low-dose VIS treatment in group 4 showed a non-significant increase, while high-dose VIS treated I group 5 (*p* < 0.01) and standard treatment group 6 (*p* < 0.01) significantly restored occludin levels. Groups 5 and 6 were comparable, showing substantial amelioration of barrier integrity (Figure 6A). IL-17, a marker of inflammatory activity, was significantly elevated in group 2 compared to group 1 (*p* < 0.001). VIS treatment reduced IL-17 expression in a dose-dependent manner, with group 4 (*p* < 0.01), group 5 (*p* < 0.001), and group 6 (*p* < 0.001) showing significant improvements compared to group 2. Groups 5 and 6 demonstrated similar reduction levels, indicating effective control of inflammation (Figure 6B). TNF-*α* expression, another key inflammatory marker, was significantly upregulated in group 2 compared to group 1 (*p* < 0.001). VIS treatment resulted in dose-dependent reductions in TNF-α levels, with group 4 (*p* < 0.01), group 5 (*p* < 0.01), and group 6 (*p* < 0.001) demonstrating significant improvements. Groups 5 and 6 exhibited comparable reductions, approaching control levels. These findings demonstrate that VIS effectively ameliorates DSS-induced colonic damage by modulating the expression of key genes associated with barrier function and inflammation (Figure 6C) and values are depicted (Table 7).

**Table 7:** Effect of visnagin in various mRNA gene expression levels in colon of different groups of mice.

Groups	Occludin gene expression in colon (A)	IL-17 gene expression in colon (B)	TNF-α gene expression in colon (C)
Group 1	0.95 ± 0.08	1.35 ± 0.11	0.97 ± 0.05
Group 2	0.43 ± 0.06^###^	6.76 ± 0.61^###^	2.17 ± 0.16^###^
Group 3	0.91 ± 0.02	1.35 ± 0.05	1.06 ± 0.15
Group 4	0.54 ± 0.03^ns^	4.90 ± 0.16**	1.52 ± 0.04**
Group 5	0.77 ± 0.05**	2.27 ± 0.10***	1.41 ± 0.16**
Group 6	0.85 ± 0.03**	2.23 ± 0.17***	1.18 ± 0.05***

^###^*p* < 0.001 G-1 Vs G-2, *p < 0.05 G-2 Vs G-4, ***p < 0.001 G-2 Vs G-4.

### 3.9 VIS mitigates the gross and histopathology against UC

**Figure 7:**
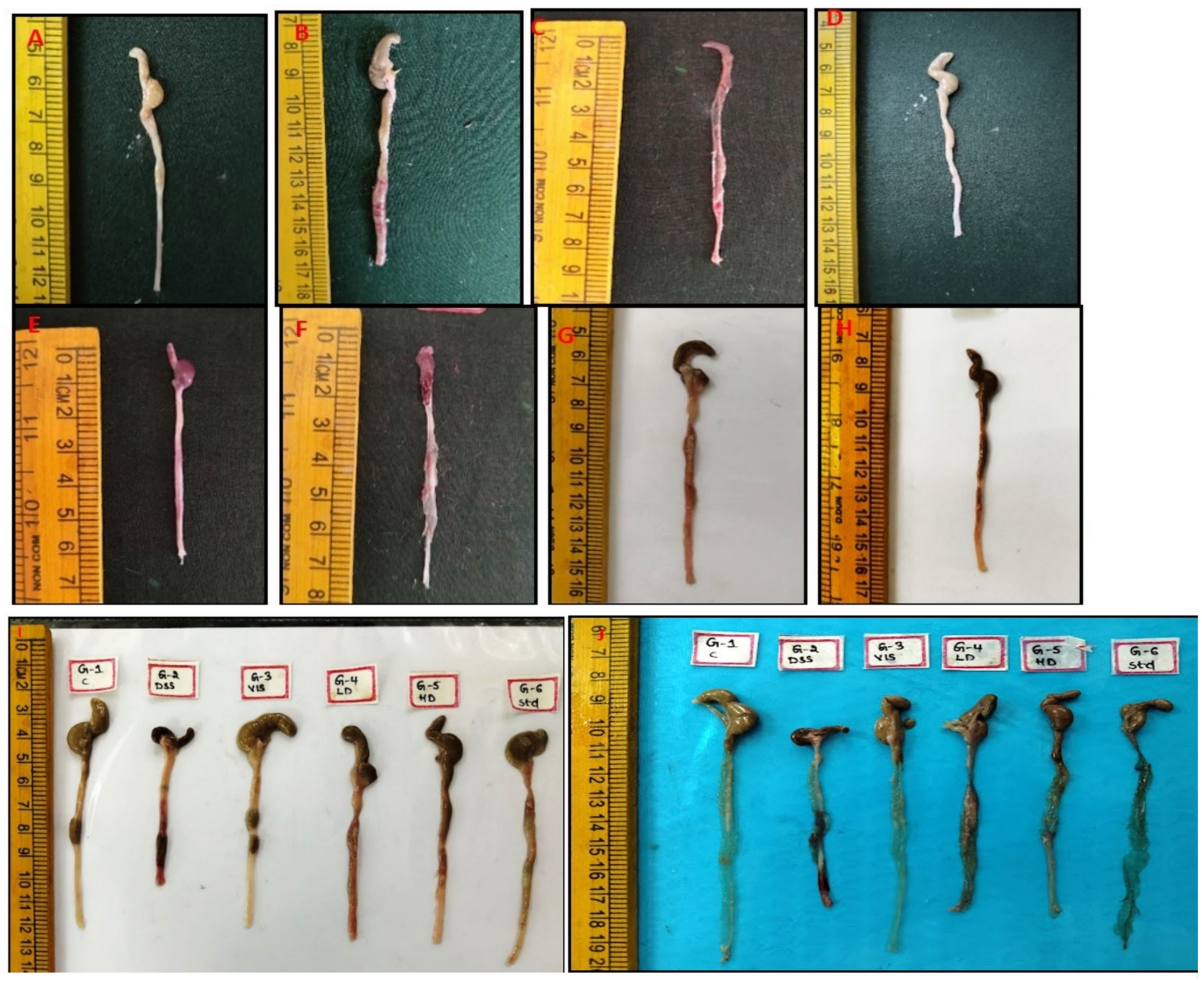
Gross appearance of colon from 6 groups. Mice showing normal appearance of colon (G-1,3, **A,D**), severe congestion and erosion in the mucosa of the colon (G-2, **B**), congestion of colon with areas of erosions and ulcers (G-2, **C**), moderate swelling and congestion of the colon tissue (G-4, **E**), moderate swelling and congestion of the colon tissue (G-4, **F**), mild congestion in the mucosa of the colon (G-5, **G**) and mild congestion of the mucosa in the colon (G-6, **H**). Colon and cut sections from different groups show variation in lengths and congestion severity **(I,J)**.

After a thorough external examination of the animals, mice were sacrificed and gross lesions were recorded. The mice in groups 1 and 3 did not showed any pathological changes in the colon and exhibited normal appearance (Figures 7A,D). In contrast, group 2 of colon showed severe congestion, erosions and ulcers on the mucosa and thickening of the wall of the colon (Figures 7B,C). The mice in group 4 revealed moderate congestion (Figure 7E) whereas mild congestion of the colon was observed in group 5 and group 6 (Figures 7F–H). The length of the colon was also markedly reduced in group 2 compared to other groups (Figures 7I,J).

**Figure 8:**
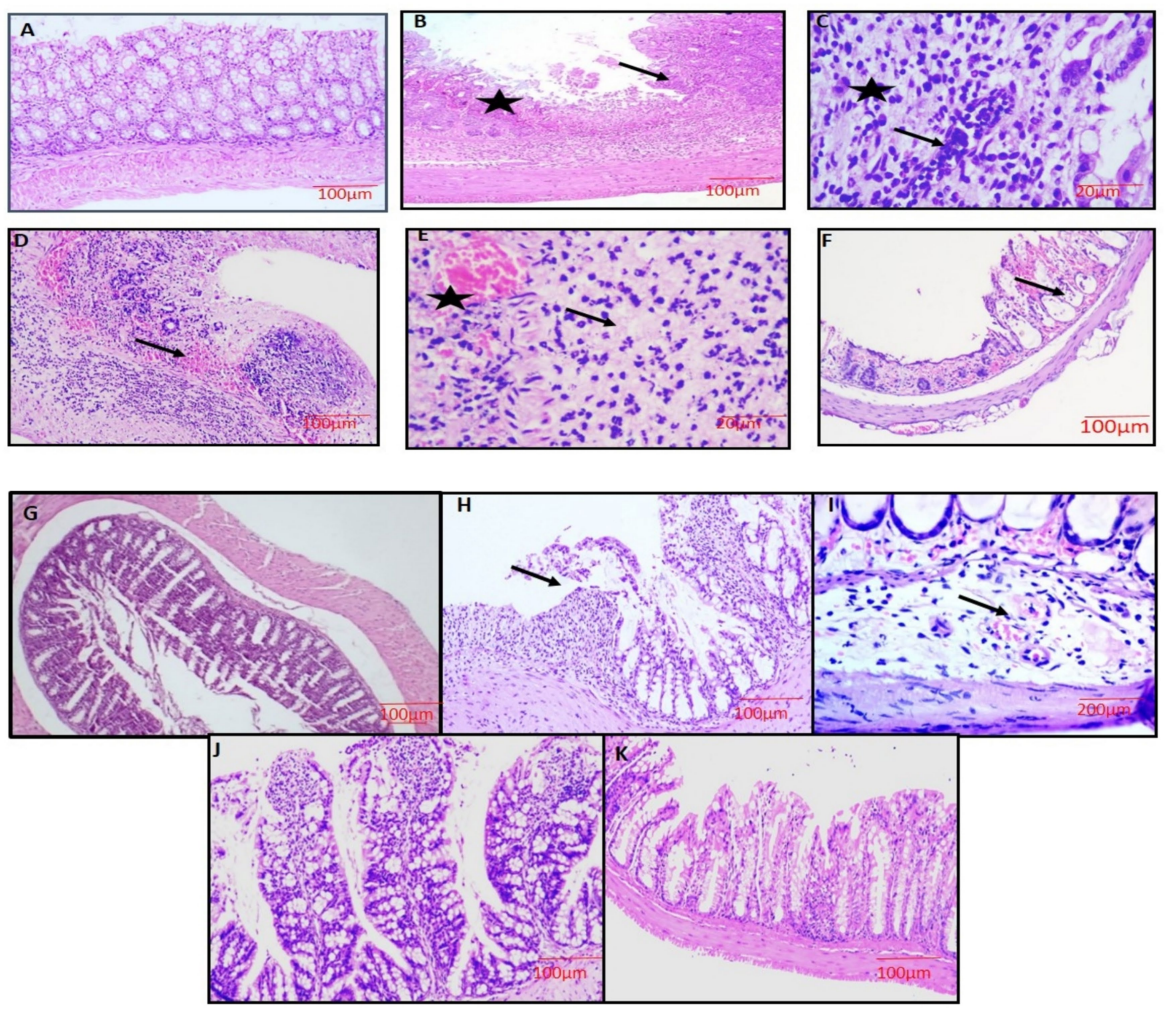
Microscopic examination of the colon showing normal architecture with all the 4 layers intact (G-1,3, **A,G**), severe necrosis of the epithelium (star),loss of colonic mucosa, disruption of crypts and ulcer formation (arrow) (G-2, **B**), showing severe loss of goblet cells (star) with severe infiltration of inflammatory cells (arrow) (G-2, **C**), severe necrosis of all layers, hemorrhages in the submucosa and lymphoid follicle and infiltration of inflammatory cells (arrow) (G-2, **D**), severe necrosis, congestion and dilatation of blood vessels (star), severe infiltration of inflammatory cells and loss of goblet cells (G-2, **E**), severe mucosal hemorrhages and cryptitis (arrow) (G-2, **F**), moderate loss of goblet cells with moderate infiltration of inflammatory cells (G-4, **H**), moderate dilatation and congestion of blood vessel in the submucosa layer with edema (G-5, **I**), mild congestion in mucosa layer and regeneration of mucosal epithelium (G-5, **J**), mucosal epithelium restoration with mild degeneration present at a few spots (G-6, **K**). H&E x100 **(A,B,D,F,G,H,J,K)**, H&E x400 **(C,E,I)**.

Microscopically, the colon from group 1 and group 3 mice displayed normal architecture with all four layers intact (mucosa, submucosa, muscularis mucosa, and serosa). The section showed mucosa with well-defined crypts, epithelium lined by goblet cells, and lamina propria (LP) comprised of loose connective tissue scattered immune cells were observed (Figures 8A,G). The sections of colon from group 2 mice showed severe damage to the colonic mucosa, loss of columnar colonocytes, and desquamation of epithelial lining with significant loss of goblet cells. There was severe crypt distortion and extensive ulcer formation (Figure 8B). Some sections revealed severe infiltration of mononuclear cells (MNCs) in all the layers (Figure 8C) and severe congestion in the sub mucosa and LP (Figure 8E). Additionally, some sections of colon showed thickened muscularis layer, severe neutrophil infiltration in all the layers and also in the lumen of crypts (cryptitis), submucosal edema, and severe congestion and hemorrhages in the mucosa of crypts and submucosa (Figures 8D,F).

In treatment groups 4 and 5, lesions were similar to group 2, but severity was markedly reduced with the restoration of goblet cells, and crypt architecture in a dose-dependent manner was noticed. The mice treated with standard drug (group 6) showed mild inflammatory changes with mucosal damage (Figures 8H–J). The degree of colon injury was observed, and lesion scoring was performed (Table 8).

**Table 8:** Histopathological scoring criteria of colon.

Groups lesions	Group-1	Group-2	Group-3	Group-4	Group-5	Group-6
Extent of inflammation	−	+++	−	++	+	+
Leucocytic infiltration	−	+++	−	++	+	+
Congestion	−	+++	−	++	+	−
Crypts damage with loss of goblet cells	−	+++	−	++	+	+
Ulcers formation	−	++	−	+	−	−
Regeneration of crypt	−	−	−	+	++	+++

−, Normal; +, Mild; ++, Moderate; +++, Severe.

### 3.10 Effect of VIS on Alcian blue and periodic acid schiff stain of colon

**Figure 9:**
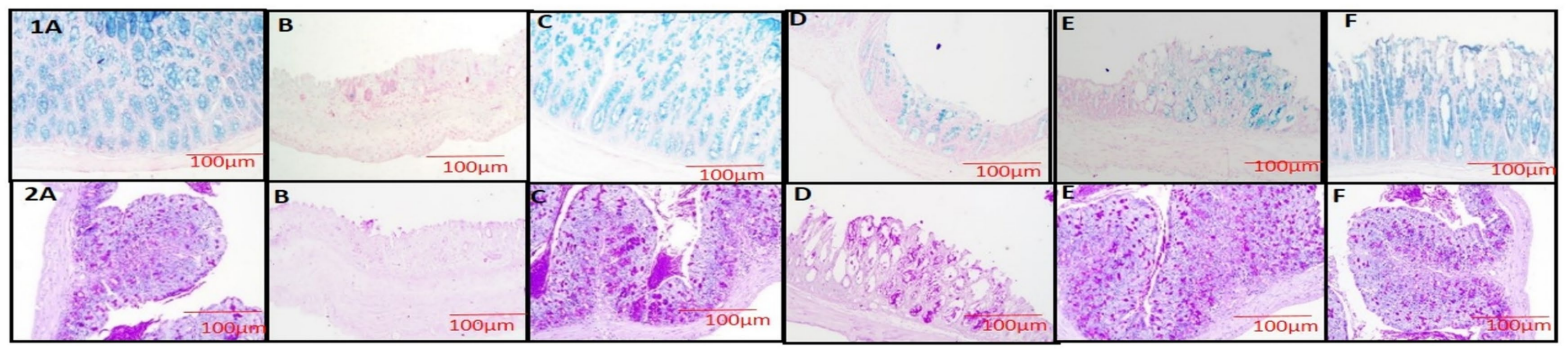
colon section showing normal distribution of acidic mucin throughout the colon (G-1,3, **1A,C**), less densely packed distribution of mucin with extensive loss of goblet cells and crypt architecture (G-2, **1B**), mild increase in mucin expressing cells (G-4, **1D**), moderate increase in mucin expressing cells (G-5, **1E**), almost normal distribution of mucin expressing cells (G-6, **1F**). Alcian blue ×100. Photomicrograph of colon section showing normal distribution of neutral and acidic mucin throughout the colon (G-1,3, **2A,C**), less distribution of mucin throughout the colon with significant loss of goblet cells and crypt architecture (G-2, **2B**), mild distribution of mucin throughout the colon (G-4, **2D**), moderate distribution of mucin throughout the colon (G-5,6, **2E,F**). PAS×100.

With Alcian blue and PAS stain, the colon sections of groups 1 and 3 exhibited uniform distribution of mucin throughout the mucosa [Figure 9 (1-A, C and 2-A, C), respectively]. In group 2, the colon sections revealed decreased mucin distribution in the mucosa, indicating damage to glands when compared to groups 1 and 3 [Figure 9 (1-B, 2-B)]. Group 4 showed more mucin-producing cells than group 2 [Figure 9 (1-D, 2-D)]. In groups 5 and 6, a marked increase in mucin-producing cells was noted in the mucosa of colon, indicating restoration of colon mucosa [Figure 9 (1-E, 2-E)] and 6 [Figure 9 (1-E,F, 2-E,F)].

### 3.11 Immunohistochemistry

**Figure 10:**
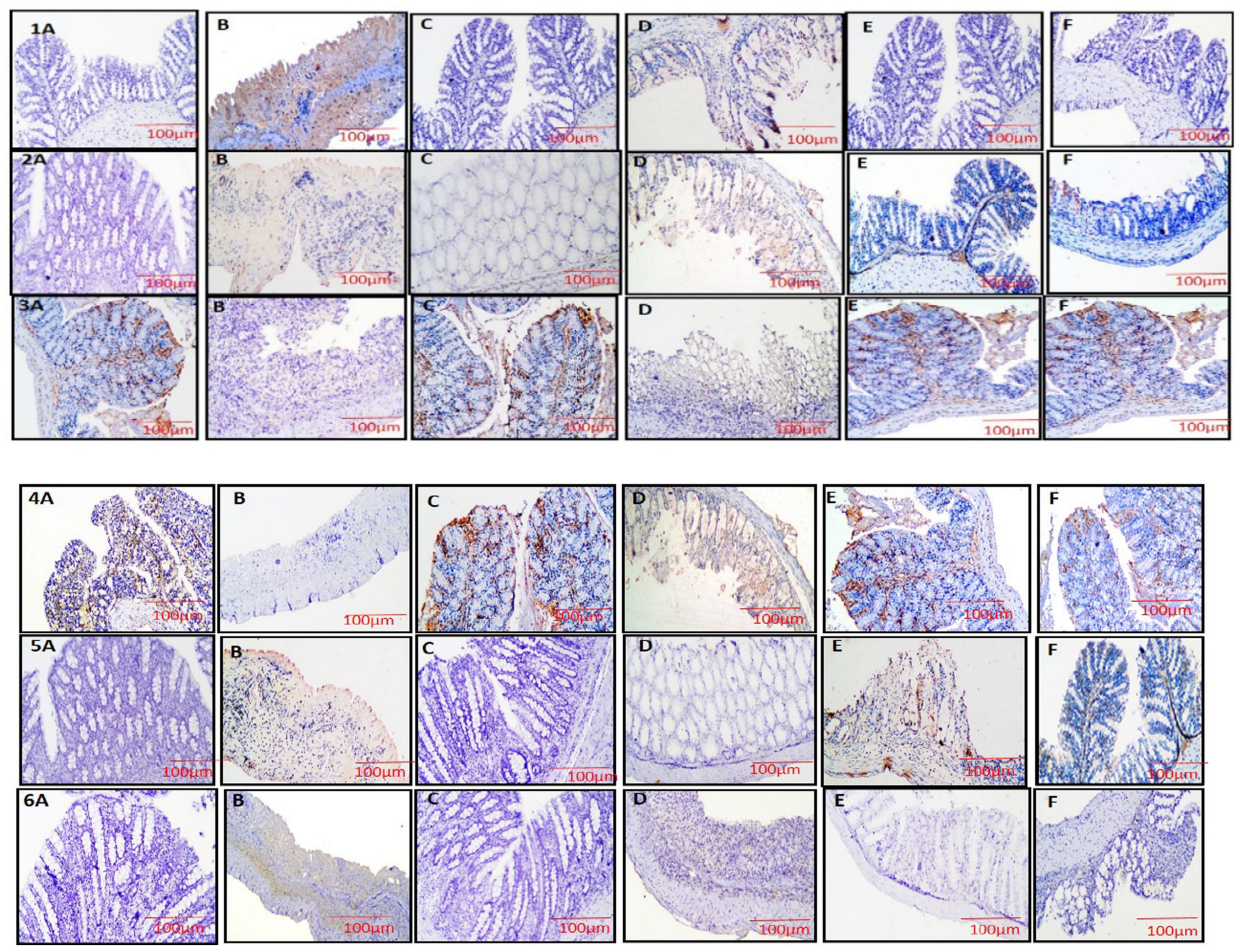
Photomicrograph of colon section showing negative immunostaining (G-1,3, **A,C**), diffuse strong immunopositive staining (G-2, **B**), moderate positive immunostaining for (G-4, **D**), mild positive immunostaining (G-5, **E**), mild to negative immunostaining (G-6, **F**). NF-κB 2. COX 5. 5. NRLP3 6. TNF-α. Photomicrograph of colon section showing positive immunostaining (G-1,3, **A,C**), negative immunopositive staining (G-2, **B**), mild positive immunostaining for (G-4, **D**), moderate positive immunostaining (G-5, **E**), moderate to positive immunostaining (G-6, **F**) IHC ×100.3. Ocludin 4. Nrf2. IHC ×100.

The study utilized IHC to analyze the expression of key inflammatory and antioxidant proteins, including NF-κB, COX-2, occludin, Nrf2, NLRP3, and TNF-*α*, in colon tissue to evaluate the anti-inflammatory and protective effects of VIS. In DSS-induced colitis (group 2), NF-κB and COX-2 showed intense immunoreactivity [Figure 10 (1,2-B)], whereas groups 4 and 5 showed reduced staining in a dose-dependent manner [Figure 10 (1,2 D,E)]. Group 6 demonstrated mild expression, comparable to the high-dose VIS group [Figure 10 (1,2 F)]. NLRP3, a marker of inflammasome activation, showed intense immunostaining in group 2 [Figure 10 (5-B)], correlating with heightened inflammation. Groups 4 and 5 demonstrated reduced NLRP3 staining [Figure 10 (5-D,E)], while group 6 showed mild expression, reflecting the anti-inflammatory effects of VIS [Figure 10 (5-A,C)]. Similarly, TNF-*α* expression was markedly elevated in group 2 but was reduced in a dose-dependent manner in VIS-treated groups [Figure 10 (6 D,E,F)]. Group 1,3 showed negative immune expression for all these parameters [Figure 10 (1,2, 5,6-A,C)],

Occludin expression, indicative of tight junction integrity, was strongly positive in control groups (1 and 3) but showed a marked reduction in group 2, highlighting compromised barrier function. VIS treatment restored occludin expression in groups 4 and 5, with mild to moderate staining, while group 6 exhibited comparable improvement. For Nrf2, a key regulator of the antioxidant response, strong immunoreactivity was observed in control groups, while DSS treatment significantly reduced its expression. VIS-treated groups displayed dose-dependent improvements, with mild to moderate Nrf2 expression, similar to group 6, suggesting an enhanced antioxidative profile [Figure 10 (3,4A–F)].

These findings collectively indicate that VIS effectively ameliorates DSS-induced colitis by modulating NF-κB, COX-2, TNF-α, and NLRP3 expression while enhancing occludin and Nrf2 levels, thereby reducing inflammation and oxidative stress and restoring intestinal barrier integrity.

### 3.12 Protective effect of VIS on ultrastructural pathology-scanning electron microscopy

**Figure 11:**
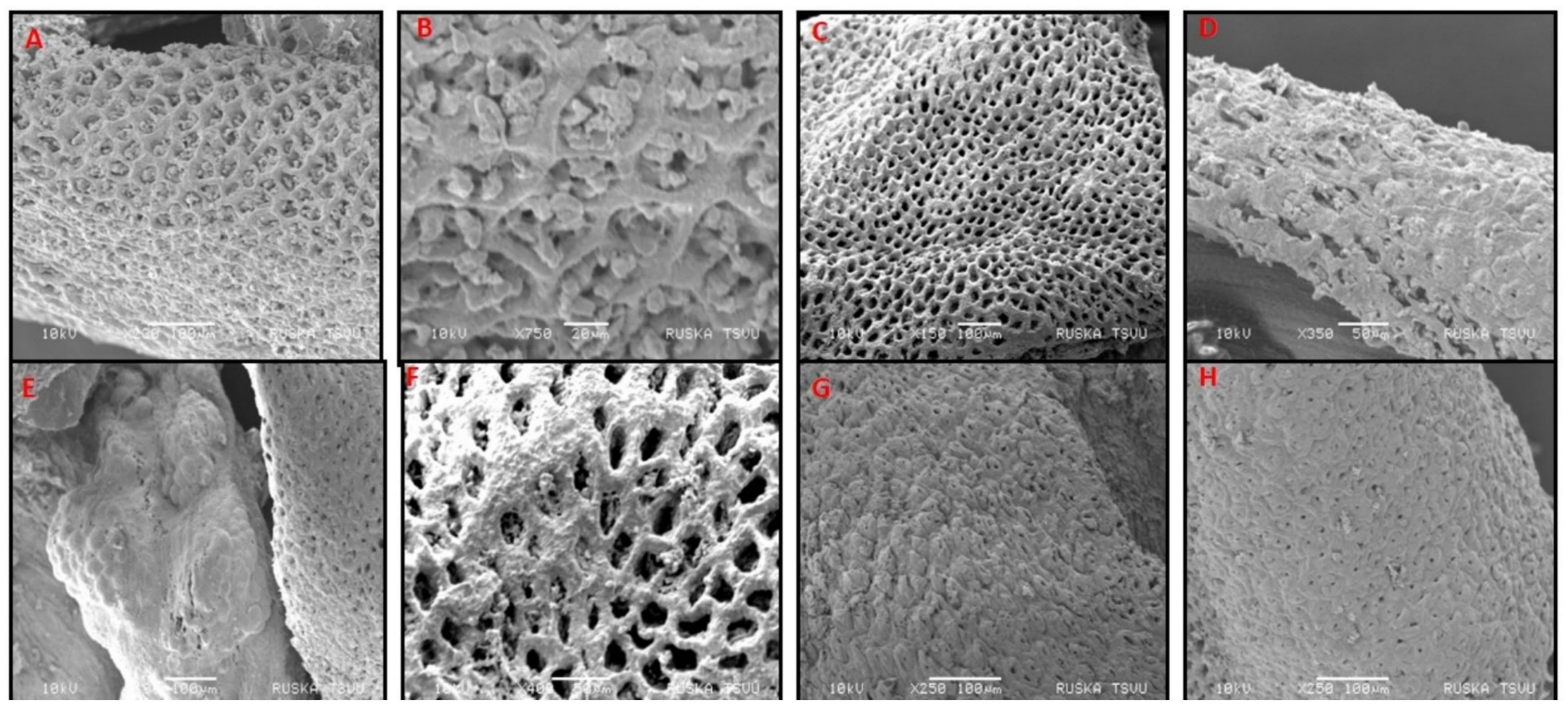
Scanning electron micrograph of colon showing normal surface of colon (G-1,3, **A,B,E**), narrow crypts opening indicates swelling of epithelial lining of crypts (G-2, **C**), necrosis of crypts (G-2, **D**), moderate dilated crypts with loss of acinar lining of crypts (G-4, **F**), mild narrowing of crypts with normal surface architecture (G-5, **G**), crypts with normal surface architecture (G-6, **H**).

Scanning electron microscopic (SEM) examination of colon revealed significant differences in surface architecture and pathological changes among the various groups. In group 1 and group 3 (Figures 11A,B,E), the colon surface appeared normal. In contrast, group 2 mice exhibited narrow crypt openings, swelling of the epithelial lining, and necrosis of crypts, indicating structural disruption (Figures 11C,D). Group 4 showed dilated crypts with loss of acinar lining of crypts (Figure 11F), whereas groups 5 and group 6 showed only mild narrowing of crypts with otherwise normal surface architecture (Figures 11G,H).

## 4 Discussion

DSS-induced experimental ulcerative colitis (UC) in mice is a widely utilized experimental model for studying pathogenesis of inflammatory bowel disease (IBD) and evaluating the therapeutic efficacy of potential interventions. This model effectively recapitulates key aspects of UC, including epithelial damage, immune cell infiltration, and excessive cytokine production. In this study, we investigated the ameliorative potential of VIS, a natural furanocoumarin with reported anti-inflammatory and antioxidant properties, in mitigating DSS-induced colonic inflammation and injury. The results revealed that VIS alleviated clinical symptoms, modulated inflammatory pathways, and improved histopathological outcomes, underscoring its potential as a therapeutic agent for UC. These findings provide valuable insights into the molecular mechanisms underlying VIS protective effects and its relevance to IBD treatment strategies.

Mice in group 2 (disease group) exhibited severe symptoms, including dullness, restlessness, weight loss, reduced food and water intake, depression, and bloody diarrhea. These effects resulted from DSS-induced negatively charged sulfated groups on the colonic epithelium, resulting in erosion and bleeding, epithelial damage, characterized by tight junction disruption (occludin, ZO-1, claudins), apoptosis, and barrier dysfunction, leading to ulcers, nutrient malabsorption, increased intestinal permeability, and mucosal ulceration. Persistent inflammation caused colon shortening due to fibrosis, wall thickening, and muscular hypertrophy. The disease progression was reflected in high DAI values and significant weight loss (30). In contrast, VIS treatment in groups 4 and 5 dose-dependently improved food intake, weight gain, colon length, and DAI scores, likely due to its anti-inflammatory and anti-apoptotic effects, enhancing epithelial repair, mucosal restoration, and reducing ROS damage. Group 6 showed slight but non-significant improvements compared to group 5, aligning with previous reports on VIS’s protective properties of anti-apoptotic and anti-inflammatory effects (12).

In patients with IBD, especially UC, oxidative stress is thought to be a major cause of tissue destruction. ROS and nitrogen metabolites play key roles in the initiation and progression of inflammatory bowel diseases (IBDs) (31). ROS plays a dual role in oxidative stress, supporting biological functions at moderate levels but causing significant cellular and molecular damage when overproduced. Excess ROS damages DNA, proteins, and lipids, increases lipid peroxidation (LPO), disrupts epithelial integrity, and increases intestinal permeability, reducing barrier function and microbial infiltration. This triggers inflammatory pathways, especially NF-κB and NLRP3, exacerbating oxidative stress and creating a vicious cycle of inflammation and ROS production (32). DSS further amplifies this process by disrupting epithelial cells, promoting dysbiosis, and activating immune cells like neutrophils and macrophages, which generate ROS production. These effects lead to mitochondrial dysfunction, tissue destruction, and severe malnutrition due to impaired intestinal absorption. Nitric oxide (NO) also contributes to inflammation and tissue injury in UC produced by iNOS in response to inflammation, NO interacts with ROS to form peroxynitrite, causing nitrosative stress, apoptosis, and mucosal barrier disruption (33). In the current study, Excess NO and LPO production in group 2 exacerbates inflammation by amplifying cytokine effects, including TNF-*α* and IL-6, further damaging the intestinal barrier and inhibiting motility.

In contrast, VIS treatment significantly reduced ROS and NO levels in groups 4 and 5 in a dose-dependent manner compared to group 2, as reported in various studies. VIS’s anti-inflammatory properties help to restore epithelial integrity, reduce oxidative stress and modulate gene expression involved in inflammation, indicating its protective effects (34).

In DSS-induced UC, oxidative stress disrupts the antioxidant defense system, leading to significant reductions in key enzymes like SOD, CAT and GSH. SOD, an essential antioxidant enzyme, converts superoxide anions into hydrogen peroxide (H₂O₂), which is further detoxified into water by CAT, preventing oxidative damage. GSH, a critical tripeptide antioxidant, scavenges ROS, facilitates DNA repair, and supports endogenous antioxidant recycling. It serves as a substrate for glutathione peroxidase (GPx), neutralizing peroxides and converting to its oxidized form, which is regenerated by glutathione reductase (35). In this study, group 2 mice exhibited significantly reduced SOD, CAT, and GSH activity compared to controls, likely due to excessive ROS production overwhelming antioxidant defenses during inflammation (36).

Recently, more studies have applied natural antioxidants to exhibit ROS scavenging signaling pathways and increase antioxidant defense capacity to inhibit pro-oxidative and pro-inflammatory cytokines in the intestinal tract, receiving favorable feedback for the treatment of IBD. VIS, a natural ingredient, exhibits potent antioxidant properties through a strong antioxidant effect by upregulating the Nuclear factor erythroid 2-related factor 2 (Nrf2) signaling pathway (12, 17).

Nrf2 is a key transcription factor that regulates antioxidant and inflammatory responses. Under stress, Nrf2 translocates to the nucleus, binding to antioxidant response elements (ARE) to upregulate antioxidant genes such as SOD and CAT, which help detoxify reactive oxygen species (ROS) (37). Nrf2 also interacts with NF-κB to maintain cellular homeostasis, modulate immune response and proteolytic enzyme activity (38), and protect intestinal membrane integrity (Jiang et al., 2024). In this study, treatment with VIS in groups 4 and 5 significantly increased SOD and CAT activity in a dose-dependent manner compared to group 2, likely through Nrf2 activation. Additionally, VIS restored GSH levels by enhancing intracellular GSH and normalizing glutathione reductase and oxidase activity, which correlated with reduced lipid peroxidation (LPO). These findings suggest that VIS ameliorates DSS-induced UC by boosting the Nrf2 signaling pathway and improving antioxidant defense in the colon, supporting its role as an ameliorative agent (38).

UC is characterized by a dysregulated colonic oxidative-inflammatory response, leading to mucosal ulceration (39). Inflammation in IBD is primarily driven by pro-inflammatory cytokines secreted by immune cells like macrophages and neutrophils, which activate the adaptive immune system (41). This further releases pro-inflammatory mediators, including TNF-*α*, IL-1*β*, and IL-6, which amplify the inflammatory response. Dysregulated TLR4/NF-κB signaling, triggered by microbiota and pathogens, produces cytokines like TNF-α and IL-6, which exacerbate inflammation in UC (40). TNF-α increases the expression of IL-1β, IL-6, and adhesion molecules and activates pathways like MAPK and NF-κB, contributing to inflammation and epithelial barrier dysfunction (41). NF-κB activation in UC promotes the transcription of inflammatory mediators and further intensifies tissue inflammation (42). Key cytokines such as IL-1β and IFN-*γ* also contribute to mucosal damage by increasing epithelial permeability and promoting cell apoptosis (42). Interleukin 6 (IL-6) performs its pro-inflammatory action using the IL-6 soluble receptor and stimulates Janus kinase/signal transducers and activators of the transcription (JAK/STAT) signaling pathway involved in the pathogenesis of UC. Once activated, STAT proteins modify the specific regulatory sequence of target genes such as iNOS and COX-2 (43). Furthermore, the imbalance between Treg and Th17 cells plays a critical role in UC, with Th17 cells promoting inflammation via IL-17 and amplifying the expression of pro-inflammatory cytokines (43). During chronic colitis, transforming growth factor (TGF-β) secretion is exacerbated, leading to elevated extracellular matrix secretion and apoptosis in epithelial cells, contributing to tissue damage (43). In response to DSS-induced colitis, pro-inflammatory cytokine levels (TNF-*α*, IL-1β, IL-6, IFN-γ, NF-κB, IL-17, and TGF-β) were significantly elevated in group 2, indicating their role in the pathogenesis of UC. VIS treatment in groups 4 and 5 reduced these cytokines in a dose-dependent manner, likely by modulating the NF-κB pathway and upregulating Nrf2 signaling, as demonstrated in immunohistochemical analysis. Thus, VIS effectively mitigates inflammation in DSS-induced UC (17, 34, 44).

The tight junction (TJ) proteins occludin, claudin, and ZO-1 are essential for maintaining the integrity of intestinal mucosal barrier, preventing pathogens from entering, and regulating intestinal permeability. Damage to these proteins is the key factor in pathogenesis of colitis. In chronic colitis induced by DSS, occludin expression is significantly reduced, indicating a compromised intestinal barrier and increased permeability (45). Visnagin (VIS) treatment demonstrated a protective effect by restoring occludin levels, thereby enhancing the integrity of the intestinal barrier. Inflammatory cytokines such as IL-17 and TNF-*α* are closely linked to the progression of colitis. IL-17, primarily produced by Th17 cells, promotes inflammation by recruiting neutrophils and macrophages while disrupting the epithelial barrier (46). Its overexpression in DSS-induced colitis was associated with dysregulation of the Treg/Th17 balance, mediated by pathways involving HIF-1α and STAT3 (43). VIS treatment significantly reduced IL-17 expression in a dose-dependent manner, likely due to its anti-inflammatory and immunomodulatory properties, which inhibit NF-κB and activator protein-1 pathways (12, 17, 47). Similarly, elevated TNF-α levels in DSS-induced colitis exacerbate epithelial damage and intestinal permeability (48). VIS effectively reduced TNF-α expression, demonstrating its strong anti-inflammatory effects via inhibiting NF-κB and MAPK signaling pathways (17). The findings suggest VIS improves occludin expression and mitigates inflammatory responses, highlighting its potential as a therapeutic agent for colitis.

RT-PCR analysis further confirmed that VIS upregulated occludin expression while significantly downregulating pro-inflammatory markers TNF-*α* and IL-17 in a dose-dependent manner. The anti-inflammatory and immunomodulatory effects of VIS are attributed to its ability to inhibit NF-κB and MAPK signaling pathways, thereby reducing epithelial injury and promoting mucosal healing in colitis (15).

In DSS-induced colitis, severe mucosal congestion and ulcers were observed, along with a significant reduction in colon length. Histopathological analysis revealed severe epithelial injury, goblet cell loss, crypt distortion, widespread ulceration, and inflammatory cell infiltration, edema, and congestion in the submucosa. These changes likely result from the sulfated polysaccharides in DSS acting as chemical toxins, damaging epithelial cells, disrupting vascular function and reducing TJ protein expression (49). These findings align with the study’s oxidative stress and inflammatory cytokine levels.

Treatment with visnagin (VIS) in groups 4 and 5 demonstrated notable ameliorative effects, with reduced congestion, partial restoration of goblet cells, improved crypt architecture and reduced inflammatory infiltration in a dose-dependent manner. The group treated with a standard drug (group 6) exhibited mild inflammatory changes and near-complete mucosal restoration. VIS exerts protective effects through anti-inflammatory, antioxidant and anti-apoptotic mechanisms, improving epithelial integrity and reducing inflammation (12).

Staining with Alcian Blue and PAS revealed decreased mucin distribution in DSS-treated group 2 due to goblet cell loss and mucosal damage (50). However, VIS treatment in groups 4 and 5 significantly restored mucin production, enhancing epithelial repair. VIS likely supports mucosal restoration by improving TJ protein expression, reducing intestinal permeability, and enhancing goblet cell recovery.

Immunohistochemistry (IHC) was employed to analyze the expression of NF-κB, COX-2, occludin, NLRP3, TNF-*α*, and Nrf2 in colon tissues. DSS treatment significantly disrupted the epithelial barrier, activating immune responses through TLR4 signaling and promoting NF-κB translocation. This activated the transcription of pro-inflammatory genes like NLRP3 and pro-IL-1β, contributing to chronic inflammation (45, 51). Upregulation of COX-2 and TNF-α in DSS-treated colons further exacerbated tissue damage by disrupting tight junctions, increasing permeability, and amplifying inflammatory responses. These changes were evident through positive immunostaining for NF-κB, NLRP3, COX-2, and TNF-α in group 2.

A decrease in occludin expression was also observed in group 2, reflecting weakened intestinal barrier integrity. Additionally, DSS inhibited Nrf2 expression, impairing the antioxidant response and increasing oxidative damage in colonic tissue.

In contrast, treatment with VIS in groups 4 and 5 reduced immunoreactivity for NF-κB, NLRP3, COX-2, and TNF-α, indicating suppressed inflammation. VIS enhanced occludin expression, improving TJ integrity and restoring intestinal barrier function. Furthermore, VIS upregulated Nrf2 signaling, evidenced by increased immunostaining, which likely contributed to its antioxidative effects and reduced oxidative stress. These findings suggest that VIS mitigates colonic damage in DSS-induced colitis by modulating NF-κB, NLRP3, and Nrf2 pathways, thereby reducing inflammation and supporting mucosal healing (17, 52).

**Figure 12:**
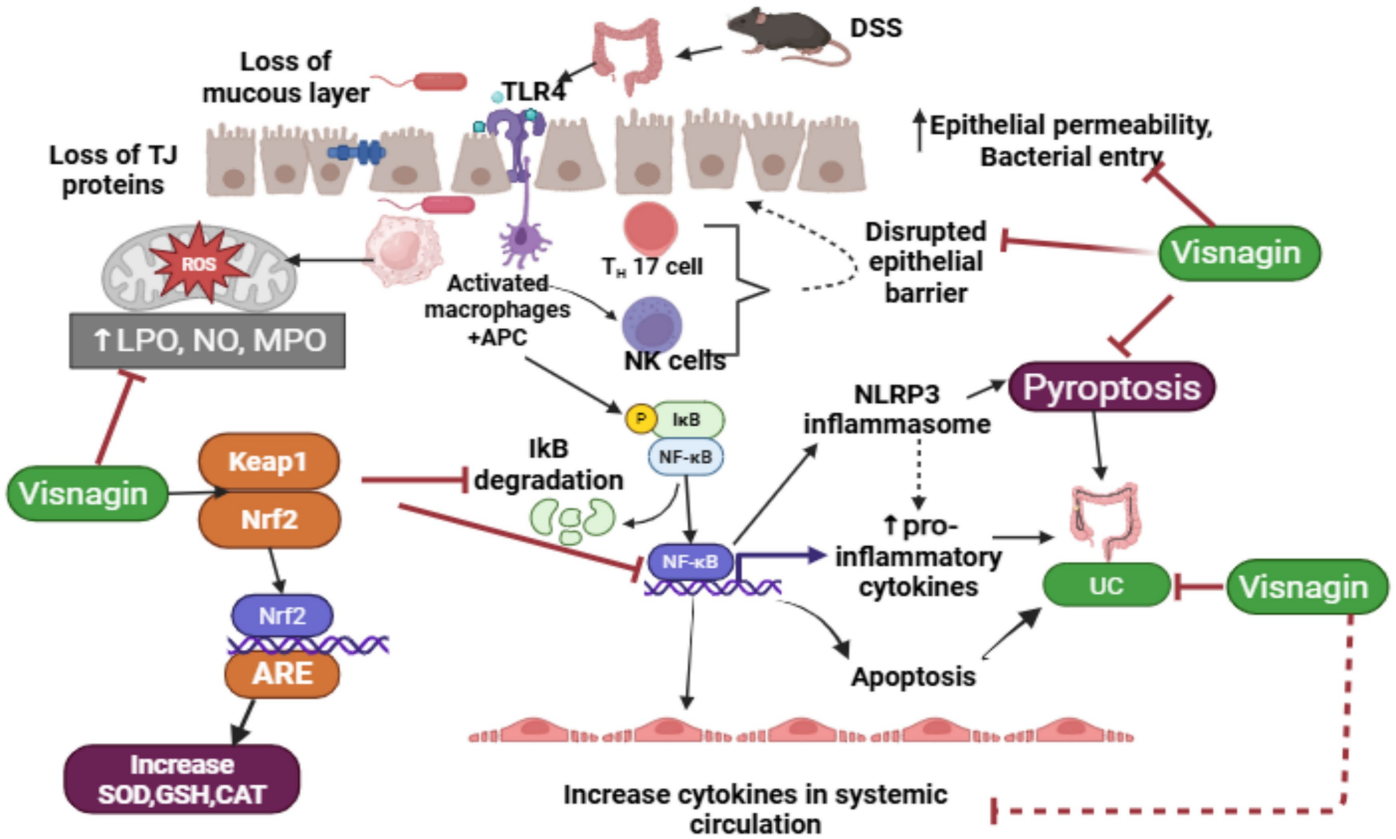
Schematic diagram of the mechanism of action of DSS-induced UC and ameliorative role of visnagin against UC.

Scanning electron microscopic (SEM) examination of the colon revealed significant differences in surface architecture and pathological changes among the various groups. In group 1 and group 3 mice, the colon surface appeared normal. In contrast, group 2 mice exhibited narrow crypt openings, swelling of the epithelial lining, and necrosis of crypts, indicating structural disruption. DSS treatment notably disrupted the microvilli structure on the colon surface, leading to disorganized, loosely arranged, and collapsed microvilli, adherence to abnormal entities, and shortened TJs, leading to UC. Similar findings were observed by Wang et al. (53). In contrast, group 4 showed dilated crypts with loss of acinar lining of crypts, and groups 5 and group 6 showed only mild narrowing of crypts with otherwise normal surface architecture. This improvement may be due to the mitigating effects of VIS against UC (Figure 12).

Dextran sulfate sodium (DSS) disrupts the intestinal mucous layer and damages tight junction (TJ) proteins, thereby compromising the epithelial barrier and increasing susceptibility to bacterial infiltration. The entry of bacteria, along with DSS, stimulates Toll-like receptor 4 (TLR4), which activates macrophages, Th17 cells, and natural killer (NK) cells. This, in turn, triggers the nuclear factor-kappa B (NF-κB) signaling pathway via IκB degradation, promoting M1 macrophage polarization and dendritic cell maturation. Activation of the NF-κB pathway also stimulates the NLRP3 inflammasome, leading to increased production of pro-inflammatory cytokines, apoptosis, and pyroptosis. These processes contribute to tissue damage and systemic inflammation, thereby exacerbating the pathogenesis of ulcerative colitis (UC).

Visnagin (VIS) exerts its protective effects by reducing oxidative stress through activation of the Nrf2 signaling pathway, which enhances the expression of antioxidant enzymes such as superoxide dismutase (SOD), glutathione (GSH), and catalase (CAT). Additionally, VIS inhibits NF-κB signaling and NLRP3 inflammasome activation, thereby reducing M1 macrophage polarization and systemic inflammation. It also restores intestinal barrier integrity by attenuating oxidative stress and inflammation, supporting epithelial repair, and preventing further bacterial translocation and tissue injury. Thus, DSS compromises intestinal integrity and provokes excessive immune activation, contributing to UC progression. VIS, through its potent antioxidant and anti-inflammatory properties, modulates key inflammatory pathways, promotes epithelial healing, and alleviates clinical symptoms of UC (Figure 12).

## 5 Limitations

Future research should focus on elucidating the precise molecular mechanisms underlying the protective effects of visnagin (VIS), optimizing its dosage for maximum efficacy, and exploring its potential synergistic interactions with other therapeutic agents. Additionally, long-term clinical studies are essential to validate its safety and effectiveness as a novel therapeutic strategy for ulcerative colitis (UC) management.

The evaluation of VIS’s pharmacokinetic properties has so far been limited to studies in mice, rats, and rabbits. Notably, VIS exhibits nonlinear pharmacokinetics, a generally undesirable characteristic in clinical settings, as it can result in a lack of dose proportionality. This nonlinearity may negatively impact both the safety and efficacy of VIS, posing a potential limitation for its development as a drug. The reported LD₅₀ for VIS is approximately 850 mg/kg in rats. Therefore, factors such as bioavailability, metabolism, and possible drug interactions should be thoroughly evaluated during the translational process to ensure safe and effective clinical application.

## 6 Conclusion

In conclusion, the present study suggests that chronic induction of DSS can cause chronic UC through increased intestinal permeability and stimulates alterations in intestinal barrier integrity and a pronounced increase in the expression of pro-inflammatory cytokines, facilitated through the upregulation of the NF-κB/NLPR3 pathway in colon. Additionally, there was a notable alteration in oxidative stress parameters attributed to downstream regulation of the Nrf-2 pathway. Moreover, VIS suppresses oxidative stress and inflammatory levels through the downstream regulation of NF-κB/NLRP3 and upstream regulation of the Nrf-2 pathway in a dose-dependent manner. Additionally, a higher dose of VIS demonstrated effects comparable to those induced by the standard drug dexamethasone. Therefore, it can be inferred that VIS can be used to manage and prevent UC.

## Data Availability

The original contributions presented in the study are included in the article/supplementary material, further inquiries can be directed to the corresponding author.
